# Seroprevalence of *Toxoplasma gondii* IgG and IgM antibodies and associated risk factors in women of child-bearing age in Njinikom, NW Cameroon

**DOI:** 10.1186/s13104-016-2206-0

**Published:** 2016-08-15

**Authors:** Elvis Chongsi Wam, Leonard Fonkeng Sama, Innocent Mbulli Ali, Walter Akoh Ebile, Lucy Agyingi Aghangu, Christopher Bonglavnyuy Tume

**Affiliations:** 1 Laboratory of Microbiology and Antimicrobial Substances, Department of Biochemistry, University of Dschang, P.O. Box 67, Dschang, Cameroon; 2 Laboratory for Public Health Research Biotechnologies, The Biotechnology Centre, University of Yaoundé 1, P.O. Box 8094, Yaoundé, Cameroon; 3 Meilleurs Accès Aux Soins de Santé Yaoundé, P.O. Box 33490, Yaoundé, Cameroon; 4 Faculty of Science, University of Dschang, PO Box 67, Dschang, Cameroon; 5 Medical Diagnostics Centre, Yaounde, Cameroon

**Keywords:** Toxoplasmosis, Seroprevalence, IgG, IgM, Antibodies, Pregnancy, HIV, Cameroon

## Abstract

**Background:**

Toxoplasmosis is a widely distributed zoonotic disease, caused by the protozoan parasite *Toxoplasma gondii*. *T.* Infections can result in stillbirths, abortions or congenital defects during pregnancy, as well as toxoplasmic encephalitis in HIV/AIDS patients. This study aimed to determine the seroprevalence and risk factors for *T. gondii* infection in women seeking antenatal and medical care in the locality of Njinikom, North West of Cameroon.

**Methods:**

We conducted a cross-sectional study from August to December 2014 consecutively enrolling 178 consenting women aged 15 to 49 years attending antenatal care or medical check-ups at the hospital. A questionnaire survey was administered to study participants and potential risk factors for *Toxoplasma* exposure sought. Venous blood was collected and serum from each participant analysed for *T. gondii* infection as evidenced by the presence of anti-*T. gondii* IgG and IgM antibodies detected using the indirect enzyme-linked immunosorbent assay (ELISA) technique. The proportion of *anti*-*T. gondii* antibody positivity calculated as the percentage of antibody seropositivity to *T. gondii* antigens. Predictors of *T. gondii* infection were analysed by univariate and multivariate regression and association with *T. gondii* seropositivity assessed. Epi-Info 3.5.4 was used for statistical analyses. A p < 0.05 was considered significant for all analyses.

**Results:**

The seroprevalence of anti-*T. gondii* antibodies (IgM or IgG) were 54.5 % (97/178). Among seropositive women, 88.7 % (86/97), 30.9 % (30/97), and 19.6 % (19/97) were respectively seropositive for IgG antibody, IgM antibody and both IgM and IgG antibodies. Among the risk factors evaluated, only the consumption of raw or undercooked meat (p = 0.02) was observed to be an independent risk factor of *T. gondii* infection. The consumption of unwashed vegetables and fruits was significant (p = 0.01) only with simple logistic regression analysis.

**Conclusions:**

Our findings suggest recent *T. gondii* exposure is high in our study population, and may constitute a significant risk factor for stillbirths, abortions or congenital defects during pregnancy in women attending antenatal care, or toxoplasmic encephalitis in those who are immunosuppressed such as in HIV/AIDS. Education and screening of HIV-positive individuals and pregnant women for *T. gondii* infection may be important primary prevention strategies in this population.

## Background

Toxoplasmosis is one of the common worldwide parasitic zoonosis, caused by the apicomplexa protozoan *Toxoplasma gondii* [1]. This parasite has cats as the definitive host, and warm-blooded animals as intermediate hosts [2]. *T. gondii* infects up to a third of the world’s population [3] and is reported to be an opportunistic parasitic infection in immune compromised hosts [4]. High prevalence of the infection has been reported among pregnant women and women of childbearing age from different parts of the world [5]. The infection occurs widely, and varies depending on social and cultural habits, geographic factors, climate, and route of transmission. It has been reported that the prevalence is higher in warm and humid areas [6].

*Toxoplasma gondii* is transmitted to humans through ingestion of oocysts in water, food or soil contaminated with cat’s faeces, or by eating raw or undercooked meat containing cysts [7–10], and women can transmit the infection through the placenta to their unborn foetus. Other infectious pathways are blood transfusion, and organs transplantation [11].

In the majority of immune competent subjects, the infection is asymptomatic [3, 12] and frequently results in the chronic persistence of cysts within the host tissues. The cysts normally lie dormant, probably for life [12]. But, in immune compromised states such as in HIV infections, subjects are at risk of developing acute toxoplasmosis due to reactivation of the organism if their CD4 cell count decreases below 200 cells/μL [13–15]. Moreover, in up to 10 % of HIV-infected immune compromised individuals, it causes cervical lymphadenopathy or ocular disease [16]. When the infection occurs in pregnant women, it can cause severe disease symptoms including toxoplasmic encephalitis, blindness, foetal abnormalities, abortion and even stillbirth [3, 17]. Toxoplasmosis has also been proven to be the cause of abortion and infertility in women [18].

In Cameroon, the few studies on toxoplasma have been limited to urban areas. For example, the seroprevalence of *T. gondii* was shown to be high among HIV/AIDS patients in the Yaoundé teaching hospital (69.9 %) [19] and pregnant women who consulted at the Department of Gynaecology in the Douala general hospital (70 %) [20]. 71.8 % prevalence was also observed among women attending antenatal care in Limbe, along the coastal region of Cameroon [21]. The study in Douala observed that the consumption of raw vegetables and untreated water were the main risk factors associated with toxoplasmosis in pregnant women [20].

The aim of this study was therefore to determine the seroprevalence of anti-*T. gondii* antibodies in HIV-infected and uninfected women of child-bearing age attending the antenatal clinic and medical check-ups and to identify the risk factors associated with *T. gondii* infection, within the rural locality of Njinikom, North West Region of Cameroon.

## Methods

### Study design

A cross-sectional study was carried out from August to December 2014 in St. Martin de Porres Catholic General Hospital Njinikom, North West Region of Cameroon.

### Study site and population

The St. Martin de Porres Catholic General Hospital Njinikom, is a private health care institution run by the Sisters of the Franciscan Congregation whose convent is located near the hospital. The hospital was created in 1963. This hospital has an HIV treatment centre, and the majority of services rendered are in the domains of gynaecology, orthopaedics and general surgery. The hospital is situated at about 48 km from Bamenda—the regional capital of the North West Region of Cameroon. About 75 % of the women in this locality depend on agriculture for their livelihoods.

Participants in this study were volunteer women of child-bearing age (HIV-infected or uninfected) attending antenatal or medical check-ups at the hospital and who duly provided consent by signing or placing their thumbprint on the consent form. Female nurses of child-bearing age working in the hospital and other carers of patients at the Hospital were also included in this study.

### Questionnaire administration and sample collection

Participants of this study were each provided with a structured questionnaire to fill. Those who could not read were assisted to fill the questionnaires by the laboratory technician or antenatal care nurse. The questionnaires contained simple closed ended questions regarding known risk factors of *Toxoplasma* exposure in addition to socio-demographic information.

Five millilitres of venous blood were collected into two tubes pre-labelled with an anonymised patient codes. The blood from the EDTA containing tubes was immediately tested for the presence of HIV antibodies using Determine (Alere Determine™ HIV-1/2) test strips according to manufacturer’s instructions. The CD4 cell count for women who were HIV positive was also measured. The second blood sample was allowed to clot completely before centrifugation at 3000 rpm for 15 min to obtain serum. Serum was separated from the clot into tightly screwed microfuge tubes and stored at −20 °C. These frozen sera were later tested for the presence of *Toxoplasma gondii* antibodies.

### Serological test for *T. gondii* antibodies

The presence of *T. gondii* antibodies in the participants’ sera was determined using an indirect Enzyme-Linked Immunosorbent Assay (ELISA). This was done using the AccuDiag™ Toxo IgM and IgG ELISA Kits (The Diagnostic Automation/Cortez Diagnostics, Inc. *Toxoplasma gondii* [Toxo] IgM and IgG Enzyme-Linked Immunosorbent Assay [ELISA]). The Toxo IgM ELISA Kits had a specificity of 100 % and a sensitivity of 100 %, while the Toxo IgG ELISA Kits had a specificity of 100 % and a sensitivity of 95.3 %. Calibrator and controls were run with each test assay. The optical densities (OD) obtained were used to calculate the cut-off calibrator value and the Immune Status Ratio (ISR). The interpretation of results was done with respect to the ISR values. For IgM, a sample with OD ≤ 0.90 was considered negative, OD ≥ 1.10 was considered positive, and OD = 0.91–1.09 was considered indeterminate. For IgG, a sample with OD < 0.90 was considered negative, OD > 1.10 was considered positive, and OD within 0.91–1.09 was considered indeterminate.

### Ethical consideration

Ethical clearance for this study was obtained from the Ethics Review and Consultancy Committee of the Cameroon Bioethics Initiative (CAMBIN) under the reference number CBI/283/ERCC/CAMBIN of August 06th, 2014. An authorisation to collect and analyse blood samples was also obtained from the St. Martin de Porres Catholic Mission Hospital Njinikom. All participants were duly informed of the study goals, procedures, potential harm and benefits, cost as well as the finality of the study. They willingly provided informed consent either by signing or placing their thumbprint on the consent form after being satisfied with responses to all questions asked the investigator. Women less than 21 years provided assent and consent from guardian was also sought for these women. Information was provided in English, French or interpreted in the local dialect by a volunteer independent of the study team. Participants’ blood samples and results were anonymised. Left over blood samples were destroyed according to hospital biosafety procedures.

### Data management and statistical analysis

Data were entered into Microsoft Excel program and then transferred to Epi Info 3.5.4 statistical program. The seroprevalence of toxoplasmosis was calculated as the proportion of serologically positive anti-*T. gondii* samples among all samples tested at 95 % confidence interval (CI). A multivariate logistic regression model was used to investigate the association between the potential risk factors for toxoplasmosis as defined by seropositivity for *Toxoplasma gondii* antibodies of any kind. Variables used in the multiple regression were selected through step-wise backward elimination using a 20 % cut-off (p to remove). The strength of associations was measured using the odds ratio (OR) at 95 % CI. The Chi-square test was used for group comparisons. Statistical significance was set at 5 % and all the associations that showed a p < 0.05 were considered significant.

## Results

A total of 350 eligible women were approached and 178 women aged 15 to 49 years provided consent for this study. The mean age of participating women was 31.13 ± 8.12 years. The majority of the women were aged between 26 and 35 years (70/178, 39.3 %). 53.9 % (96/178) of the women were married, and 6.2 % (11/178) never went to school. Among the study participants, only 5.6 % (10/178) had cats in their homes, while 30.3 % (54/178) frequently come in contact with cats because of neighbours owning cats. A total of 59.6 % (106/178) consumed raw or undercooked meat. 5.1 % (9/178), 65.7 % (117/178), and 73.1 % (130/178) women consumed raw milk, raw unwashed vegetable or fruits, and untreated drinking water respectively. Regarding the participants residential area, 5.1 % (9/178) of the study participants lived in an urban area, while 94.9 % (169/178) lived in a rural area. Those women who lived in cemented and non-cemented floor houses were 38.2 % (68/178), and 61.8 % (110/178) respectively. Only 1.7 % (3/178) knew about toxoplasmosis (Table 1). Out of the 178 women recruited in this study, 50.6 % (90/178) were HIV-positive, 49.4 % (88/178) were HIV-negative, 50.6 % (90/178) were pregnant and 49.4 % (88/178) were not pregnant. Of the 90 HIV-positive participants only 3.3 % (3/90) had a CD4 count less than 200 cells/mm^3^ and 84.5 % (76/90) HIV-infected participants lived on HAART (Table 1).

**Table 1:** General characteristics of study participants

Characteristic	Number (%)
*Age category*	
15–25	53/178 (29.8)
26–35	70/178 (39.3)
36–49	55/178 (30.9)
*Marital status*	
Married	96/178 (53.9)
Divorced	8/178 (4.4)
Single	62/178 (34.8)
Widow	12/178 (6.7)
*Level of education*	
None	11/178 (6.2)
Primary	87/178 (48.9)
Secondary	63/178 (35.4)
Higher	17/178 (9.5)
*Have cats at home*	
Yes	10/178 (5.6)
No	168/178 (94.4)
*Contact with cats*	
Yes	54/178 (30.3)
No	124/178 (69.7)
*Consume raw or undercooked meat*	
Yes	106/178 (59.6)
No	72/178 (40.4)
*Consumed raw milk*	
Yes	9/178 (5.1)
No	169/178 (94.9)
*Consumed raw unwashed vegetable or fruits*	
Yes	117/178 (65.7)
No	61/178 (34.3)
*Consumed untreated drinking water*	
Yes	130/178 (73.1)
No	48/178 (26.9)
*House floor type*	
Cemented	68/178 (38.2)
Not cemented	110/178 (61.8)
*Knowledge of toxoplasmosis*	
Yes	3/178 (1.7)
No	175/178 (98.3)
*Residential area*	
Urban	9/178 (5.1)
Rural	169/178 (94.9)
*Pregnant*	
Yes	90/178 (50.6)
No	88/178 (49.4)
*HIV positive*	
Yes	90/178 (50.6)
No	88/178 (49.4)
*HAART therapy*	
Yes	76/90 (84.5)
No	14/90 (15.5)
*CD4 cell count (cells/mm* ^*3*^ *)*	
>500	40/90 (44.5)
200–500	47/90 (52.2)
<200	3/90 (3.3)

### Prevalence of anti-*Toxoplasma* antibodies in the study population

The combined seroprevalence of anti-*T. gondii* antibodies among the 178 women of child-bearing age in our study area was calculated to be 54.5 % (97/178). Among the seropositive women, 86 were seropositive for IgG antibodies, 30 were seropositive to IgM antibodies, and 19 were seropositive for both IgG and IgM antibodies giving a prevalence of 48.3, 16.9 and 10.7 % respectively (Table 2).

**Table 2:** Seroprevalence of antibodies to *Toxoplasma gondii* according to pregnancy and HIV status

	HIV status	Pregnant	Non pregnant	Total	Grand total
IgG^+^	HIV-positive	7/17 (41.2 %) (18.4–67.1)	36/73 (49.3 %) (37.4–61.3)	43/90 (47.8 %) (37.1–58.6)	86/178 (48.3 %) (40.8–55.9)
	HIV-negative	37/73 (50.7 %) (38.7–62.6)	6/15 (40 %) (16.3–76.7)	43/88 (48.9 %) (38.1–59.7)	
IgM^+^	HIV-positive	2/17 (11.8 %) (1.6–36.4)	14/73 (19.2 %) (10.9–30.1)	16/90 (17.8 %) (10.5–27.2)	30/178 (16.9 %) (11.7–23.2)
	HIV-negative	10/73 (13.7 %) (6.8–23.7)	4/15 (26.7 %) (7.8–55.1)	14/88 (15.9 %) (9.0–25.2)	
IgG^+^ and IgM^+^	HIV-positive	1/17 (5.9 %) (0.1–28.7)	10/73 (13.7 %) (6.8–23.7)	11/90 (12.2 %) (6.3–20.8)	19/178 (10.7 %) (6.6–16.2)
	HIV-negative	7/73 (9.6 %) (3.9–18.8)	1/15 (6.7 %) (0.2–31.9)	8/88 (9.1 %) (4.0–17.1)	
IgG^+^ or IgM^+^	HIV-positive	8/17 (47.1 %) (22.9–72.2)	40/73 (54.8 %) (1.5–13.4)	48/90 (53.3 %) (42.5–63.9)	97/178 (54.5 %) (46.9–70.0)
	HIV-negative	40/73 (54.8 %) (42.7–66.5)	9/15 (60 %) (32.3–83.7)	49/88 (55.7 %) (44.7–66.3)	

^**+**^Positive: Percentages calculated with respect to totals in each sub category. For example, we found that 41.2 % of women who were HIV positive and pregnant were seropositive to IgG antibody to T. gondii

Table 2 also illustrates that the seroprevalence of antibodies to *T. gondii* was slightly higher in HIV-negative (55.7 %, [49/88]), than in HIV-positive (53.7 % [48/90]) participants (p > 0.05). Seroprevalence of anti-*T. gondii* IgG antibody was 47.8 % (43/90) in HIV-positive participants, and slightly lower than in HIV-negative participants (48.9 %, [43/88]). On the other hand, 12.2 % (11/90) of HIV-positive against 9.1 % (8/88) of HIV-negative women were seropositive to both IgG and IgM antibodies to *T. gondii*. Similarly, a higher seroprevalence of IgM antibody to *T. gondii* was found in HIV-positive (17.8 %, [16/90]), than in HIV-negative (15.9 %, [14/88]) women. A total of 55.7 % (49/88) non-pregnant women were seropositive to anti-*T. gondii* antibodies. This was slightly higher than the 53.3 % (48/90) pregnant women also seropositive to anti-*T. gondii* antibodies (Table 2).

### Risk factors for Toxoplasma seropositivity

As shown in Table 3, simple logistic regression analysis with suspected variable indicated that the consumption of raw or undercooked meat (p = 0.01) and the consumption of raw unwashed vegetables and fruits (p = 0.01) were predictors of toxoplasmosis seropositivity irrespective of the antibody type. However, results of multiple logistic regression analysis of selected variables as shown in Table 4 showed that only the consumption of raw or undercooked meat (p = 0.02) was an independent predictor of toxoplasmosis seropositivity irrespective of the antibody type. No significant association was found between *Toxoplasma gondii* seropositivity and HIV status (p = 0.18), cat ownership (p = 0.91), contact with cats (p = 0.31) and residential area (p = 0.16).

**Table 3:** Simple logistic regression with suspected variables

Variable	No of subjects tested (n, %), N = 178	Odd ratio (95 % CI)	p value
*Age (years)*			0.67
15–21	20 (11.2)	1.2	
>21	158 (88.8)	(0.48–3.11)	
*Education*			0.53
Yes	167 (93.8)	0.67	
No	11 (6.2)	(0.19–2.37)	
*Marital status*			0.84
Married	96 (53.9)	1.06	
Unmarried	82 (46.1)	(0.59–1.92)	
*House floor type*			0.79
Cemented	68 (38.2)	1.0848	
Not cemented	110 (61.8)	(0.5922–1.9873)	
*Residential area*			0.81
Rural	9 (5.1)	1.1782	
Urban	169 (94.9)	(0.3058–4.5397)	
*Pregnancy status*			0.25
Yes	90 (50.6)	0.8522	
No	88 (49.4)	(0.6469–1.1225)	
*HIV status*			0.88
Yes	90 (50.6)	0.9574	
No	88 (49.4)	(0.5318–1.7238)	
*Cat ownership*			0.91
Yes	10 (5.6)	0.9310	
No	168 (94.4)	(0.2599–3.3352)	
Contact with cats			0.31
Yes	54 (30.3)	1.3920	
No	124 (69.7)	(0.73–2.65)	
*Consumption of raw or undercooked meat*			0.01*
Yes	106 (59.6)	2.18	
No	72 (40.4)	(1.17–4.02)	
Consumption of raw unwashed vegetables or fruits			0.01*
Yes	117 (65.7)	2.08	
No	61 (34.3)	(1.11–3.89)	
*Consumption of raw milk*			0.81
Yes	9 (5.1)	1.1782	
No	169 (94.9)	(0.3058–4.5397)	

* p < 5 %: statistical significance; OR > 1 and OR < 1: association of variable with toxoplasmosis

**Table 4:** Multiple logistic regression of selected variables

Variables	Adjusted odd ratio (95 % CI)	p value
*HIV status*	0.74 (0.40–1.49)	0.18
Yes		
No		
*Consumption of raw or undercooked meat*	2.11 (1.09–4.06)	0.02*
Yes		
No		
*Consumption of raw unwashed vegetables or fruits*	1.85 (0.96–3.62)	0.07
Yes		
No		
*Residential area*	2.90 (0.65–13.0)	0.16
Rural		
Urban		

* p < 5 %: statistical significance; OR > 1 and OR < 1: association of variable with toxoplasmosis

## Discussion

Toxoplasmosis is a curable but potentially fatal disease [22]. Over the years, infection with the protozoan parasite *Toxoplasma gondii* has been proven to be one of the most common parasitic infections of man and other warm-blooded animals [2]. In this study, we sought to investigate *Toxoplasma* infection, as evidenced by anti-*Toxoplasma* antibodies in the serum of women of child bearing age in a rural locality in Cameroon. Our findings revealed that the seroprevalence of anti-*T. gondii* antibodies among the women of child-bearing age in this study was 54.5 %. This prevalence was similar to that obtained among women of child-bearing age in Timis, Romania (57.6 %) [23], but far lower than that found in women of child-bearing age in Central Ethiopia (81.4 %) [24]. This lower prevalence could be attributed to a lower cat density (10/178, Table 1) and consequent oocysts shedding and a lower consumption of unpasteurised milk in our study area. The seroprevalence obtained in this study is also far lower than that obtained in other parts of Cameroon. Indeed, a prevalence of 77.1 % was observed in pregnant women in Yaoundé [25], 70 % in pregnant women in Douala [20], 69.9 % in HIV/AIDS patients in Yaoundé [19], while a prevalence of 71.8 % was observed among pregnant women in Limbe [21]. These geographic differences in prevalence rates may be explained by differences in rural/urban setting since exposure and the main sources of infection appear to be the same. This might explain differences in risk factors of *Toxoplasma* seropositivity as well as climatic changes. Our study population was mainly from a rural area.

The seroprevalence of anti-*T. gondii* IgG antibody in HIV-positive participants was 47.8 % (43/90). This was not significantly different from the 48.9 % (43/88) obtained in the HIV-negative group in this same study population (*X*^2^ = 1.3632, p = 0.1728). This results were similar to the 42.1 % (56/133) obtained among HIV/AIDS patients in the university teaching hospital in Yaoundé [19], although the number of HIV patients in our study was smaller. Similarity in the prevalence of *Toxoplasma* seropositivity among HIV-infected or uninfected women in our study conforms to what has been reported by researchers in Malaysia [26], United States of America [27], and Ethiopia [28]. However, another study in Ethiopia showed a significantly higher prevalence of anti-*Toxoplasma gondii* antibodies (87.4 % vs 70.29 %, P = 0.003) in HIV-positive pre-antiretroviral therapy (pre-ART) individuals than in HIV-negative blood donors [10]. Indeed, in our study, up to 84.5 % (76/90) of the HIV positive study participants were on highly active antiretroviral therapy (HAART), and only three participants had CD4 counts less than 200cells/mm3 of whole blood. This indicate the benefit of HAART treatment which helps to decrease the viral load and improve CD4+ cell counts and consequently boosts the immune system of the host, with the added benefit of reducing the likelihood of reactivation of latent *T. gondii* infections [1, 29, 30]. We found a non-significant higher IgM antibodies in HIV positive women compared to HIV negative women, further confirming this assertion.

The seroprevalence of anti-*T. gondii* antibodies was higher in non-pregnant women (55.7 %, [49/88]) compared to pregnant women (53.3 %, [48/90]) irrespective of HIV status, although not significantly different. Among the 90 pregnant women enrolled in this study, 48.9 % (44/90) and 13.3 (12/90) were positive for anti-*T. gondii* IgG and IgM antibodies respectively. These prevalence values were significantly low compared to 71.8 and 67.6 % respectively obtain among pregnant women in the Limbe health district in the South West Region of Cameroon [21].

The consumption of raw or undercooked meat [p = 0.0184] was significant a risk factor associated with *T. gondii* infection in the univariate and multivariate analysis (Tables 3a and 4). In addition, consumption of unwashed vegetables or fruits was observed to be significantly associated with toxoplasma seropositivity in the univariate analysis, although it was only marginally significant in the multivariable analysis Table 4. Similar results have been observed in studies done in Mexico [31], Ethiopia [10], and Sudan [32], whereas studies done in Douala-Cameroon [20], and Thailand [33], showed a significant association between *T. gondii* infection and consumption of untreated water only. Although domestic cats are probably the major source of contamination [1, 34], cat ownership and contact with cats were not found to be significantly associated with *T. gondii* infection [9, 13]. Indeed, only 6 % of the total study population reported having cats at home. Sporulated oocysts survive in moist soil for months to years [35]. Though no significance association was found between *T. gondii* infection and untreated water, it is likely that oocysts might have been present in some of the untreated water sources in our study area. Poor sanitation methods with the poor quality water used to wash raw meat at abattoirs or vegetables sold in the market, might have led to their contamination with oocysts.

Our study however should be interpreted with some caution. Our sample size was small and conclusions from the present study must be measured. It is likely that we may obtain different results with increased sample size. The number of equivocal results obtained using the selected ELISA technique in some cases was high, and difficult to interpret with the absence of reference testing facilities for toxoplasmosis in or around our study area. This could affect our results by increasing or decreasing the observed prevalences. Secondly, we observed an increase in risk of seropositivity with stage in pregnancy, but we did not follow up those women who were seropositive to study delivery outcomes or health status of their children. Thirdly, we may have obtained a different result if we considered sampling only women of child bearing age at the antenatal service. However, less than 30 % of our study population was sampled among those who came for medical check-up at the hospital, making our results more likely to reflect what we desired to investigate.

## Conclusions

*Toxoplasma gondii* infection appears to be a public health concern in Njinikom, with a global antibody seroprevalence of 54.5 % among women of child bearing age attending antenatal or outpatient medical care in our study area. In this study, recent infections were found in 16.9 % of the study population while up to 10.7 % of patients were reactivating and there was no difference between pregnant and non-pregnant or HIV negative or HIV positive women. Consumption of raw or undercooked meat and of unwashed vegetables were observed to be risk factors for *T. gondii* infection among women of childbearing age in the locality of Njinikom, North West Region of Cameroon. Only 1.7 % (3/178) of the study participants knew about toxoplasmosis. Education on toxoplasmosis during antenatal care or in HIV treatment centres and screening for recent *Toxoplasma* exposure may be strategies for primary prevention of toxoplasmosis and its devastating outcomes in pregnancy or among HIV patients.
